# Dielectric properties of almond kernels associated with radio frequency and microwave pasteurization

**DOI:** 10.1038/srep42452

**Published:** 2017-02-10

**Authors:** Rui Li, Shuang Zhang, Xiaoxi Kou, Bo Ling, Shaojin Wang

**Affiliations:** 1 College of Mechanical and Electronic Engineering, Northwest A&F University, Yangling, Shaanxi 712100, China; 2 Department of Biological Systems Engineering, Washington State University, Pullman, WA 99164-6120, USA

## Abstract

To develop advanced pasteurization treatments based on radio frequency (RF) or microwave (MW) energy, dielectric properties of almond kernels were measured by using an open-ended coaxial-line probe and impedance analyzer at frequencies between 10 and 3000 MHz, moisture contents between 4.2% to 19.6% w.b. and temperatures between 20 and 90 °C. The results showed that both dielectric constant and loss factor of the almond kernels decreased sharply with increasing frequency over the RF range (10–300 MHz), but gradually over the measured MW range (300–3000 MHz). Both dielectric constant and loss factor of almond kernels increased with increasing temperature and moisture content, and largely enhanced at higher temperature and moisture levels. Quadratic polynomial equations were developed to best fit the relationship between dielectric constant or loss factor at 27, 40, 915 or 2450 MHz and sample temperature/moisture content with R^2^ greater than 0.967. Penetration depth of electromagnetic wave into samples decreased with increasing frequency (27–2450 MHz), moisture content (4.2–19.6% w.b.) and temperature (20–90 °C). The temperature profiles of RF heated almond kernels under three moisture levels were made using experiment and computer simulation based on measured dielectric properties. Based on the result of this study, RF treatment has potential to be practically used for pasteurization of almond kernels with acceptable heating uniformity.

Almonds are California’s top agricultural export and largest tree nuts based on total production areas and export values. In 2013, the production of almonds in California was approximately 1.81 million tons^1^. About 30–40% of the almonds are sold for domestic use, and the rest is for export. A major concern for raw almonds is possible contamination of *Salmonella Enteritidis* PT 30 when almonds fall to the ground after mechanical shaking. The almond shell is the first point to contaminating bacteria, and removing the shell can reduce 1.75-log CFU/g *Salmonella*^2^, but result in cross-contamination in the huller/sheller facility and recontamination of *Salmonella Enteritidis* PT 30 on the almond kernel. Outbreaks of *Salmonella Enteritidis* PT 30 associated with raw almonds accidentally occurred in USA and Canada in 2000 to 2001 and 2004^3^,^4^. The US Department of Agriculture (USDA) and the Almond Board of California (ABC) mandate that all almonds sold must be pasteurized to reduce pathogens in a reasonably acceptable level^5^.

Dielectric heating, which includes radio frequency (RF) and microwave (MW) treatments, has been proposed for pathogen control in agricultural commodities due to rapid volumetric heating and quality maintenance^6^,^7^,^8^,^9^,^10^. For the in-shell almond, RF heating has effectively been used for pasteurizing *Salmonella* PT 30^6^,^11^,^12^. However, for the almond kernel, there is little information about using RF and MW energy for pasteurizing *Salmonella* PT 30. In developing such pasteurization treatments, dielectric properties of almond kernels are essential to understand the interactions of the product with electromagnetic energy. Penetration depth of electromagnetic wave into samples needs to be estimated for industrial applications.

Thermal resistance of pathogens is lower in moist environment than dry environment. For example, after increasing water activity from 0.601 to 0.946 in almonds, *D*_68°C_ value of *Salmonella* PT 30 decreased from 6.97 to 0.42 min^13^. Therefore, for reducing the thermal resistance of *Salmonella* PT 30, the moisture content of almond kernel should be raised before dielectric heating. Therefore, the dielectric properties of almond kernels as influenced by moisture content need to be explored.

Dielectric properties of agricultural products influence the absorption and dissipation of electromagnetic energy when they are subjected to RF or MW heating and are generally expressed in terms of relative complex permittivity, *ε* = *ε*′ − *jε*″. The real part, *ε*′, known as the relative dielectric constant, represents a material’s ability to store the applied electrical energy. The imaginary part, *ε*″, known as the relative dielectric loss factor, measures the dissipation of applied electric energy in the form of heat^14^,^15^.

Dielectric properties of agricultural products have been studied over different frequency, temperature and moisture ranges for drying^16^,^17^,^18^,^19^,^20^, pest control^21^,^22^ and pasteurization^23^,^24^,^25^,^26^. For example, Zhang *et al*.^17^ reported that the dielectric properties of ground peanut kernels decreased with increasing frequency and increased with increasing moisture content and temperature. Gao *et al*.^23^ studied the dielectric properties of almond shells over the frequency range of 10–1800 MHz and temperatures between 20 and 90 °C with moisture content from 6% to 36% w.b. Dielectric properties of almond and walnut kernels at the temperature between 20 and 60 °C with moisture content of 3% in the range of 1–1800 MHz were reported by Wang *et al*.^27^. Up to now, dielectric properties of almond kernel over different moisture contents at RF and MW ranges are still not available for dielectric pasteurization.

The objectives of this study were to (1) determine the dielectric properties of almond kernel over the frequency range from 10 to 3000 MHz, five moisture contents (4.2–19.6% w.b.), and the eight temperatures between 20 to 90 °C, (2) provide the empirical equations describing dielectric properties of almond kernels as a function of moisture content and temperatures at interested frequencies (27.12, 40.68, 915 and 2450 MHz), (3) evaluate the penetration depth of electromagnetic energy into the almond kernels at four industrial application frequencies, and (4) confirm the RF heating rate of almond kernels at three moisture content levels using experiment and computer simulation.

## Results

### Density, moisture content and water activity of almond kernels

Table 1 showed that as the moisture content of almond kernel increased, the true density was within 1.039 to 1.060 g/cm^3^, which are in agreement with the trend of peanut and pistachio kernels reported by Ling *et al*.^16^ and Zhang *et al*.^17^. The true density increased slightly since the increased weight of almond kernels was higher than its volume expansion on moisture gain. Because the density changes in almond kernels were negligible, the density effect of the almond kernels was not considered in this study.

**Figure 1:**
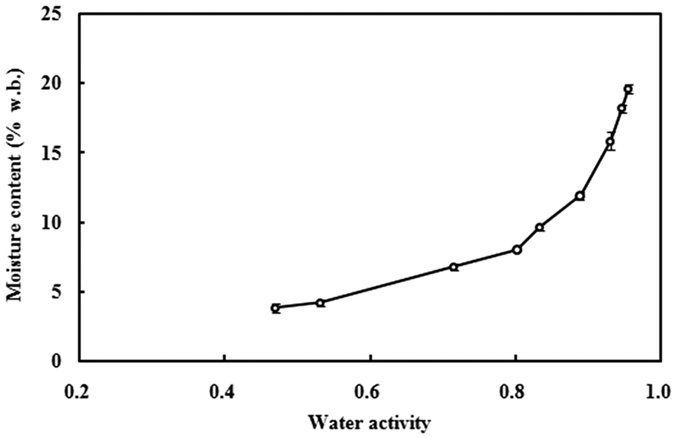
Moisture sorption isotherm of almond kernel samples at 25 °C.

Moisture sorption isotherm of almond samples at 25 °C is shown in Fig. 1. The water activity of samples increased with increasing moisture content. For almond kernel, each moisture content level corresponded to a fixed water activity. Therefore, water activity dependent dielectric properties could be used to develop practical applications for quickly estimating the moisture content of almond kernel.

### Frequency-dependent dielectric properties of almond kernels

**Figure 2:**
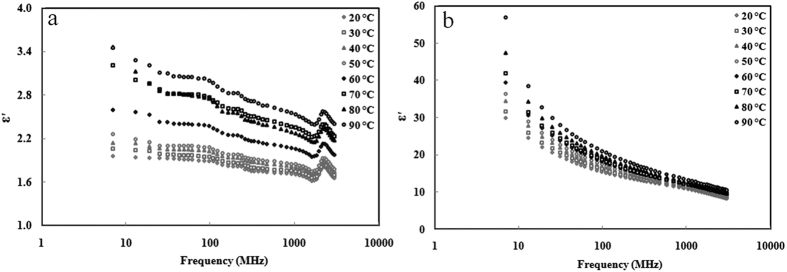
Frequency- dependent dielectric constant (ε′) of almond kernels at 20(◇), 30(□), 40(△), 50(○), 60(◆), 70(■), 80(▲) and 90 °C (●) with moisture content of 4.2% (**a**), and 19.6% w.b. (**b**).

**Figure 3:**
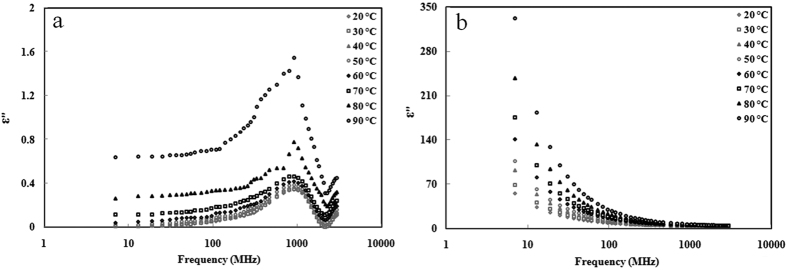
Frequency-dependent dielectric loss factor (*ε*″) of almond kernels at 20(◇), 30(□), 40(△), 50(○), 60(◆), 70(■), 80(▲) and 90 °C (●) with moisture content of 4.2% (**a**), and 19.6% w.b. (**b**).

Figures 2 and 3 showed the frequency dependent dielectric constant and loss factor of almond kernels at the temperature range from 20 to 90 °C with moisture contents of 4.2% and 19.6% w.b. At the moisture content of 4.2% w.b., all the dielectric constant and loss factor values were less than 4 at all measured temperatures due to low moisture and high fat contents in the almond kernels. The dielectric constant of almond kernels decreased with increasing frequency when the frequency was below 1600 MHz, and then increased above 1600 MHz, the peak value for dielectric constant was between 2000 and 2800 MHz. The loss factor peaked in the range between 500–1000 MHz. This phenomenon was completely disappeared when the moisture content was higher than 11.9% w.b. At the moisture content of 19.6% w.b., both dielectric constant and loss factor decreased with increasing frequency at all temperatures. Temperature and moisture content of samples had a significant effect on the frequency-dependent dielectric constant and loss factor, especially within the RF ranges. For example, when the frequency increased from 10 to 200 MHz, the dielectric constant and loss factor of samples decreased from 56.94 to 17.86 and 332.64 to 16.71 at 90 °C, and from 29.84 to 13.68 and 55.09 to 5.26 at 20 °C, respectively. Moreover, at higher moisture contents of samples, the effect of frequency on dielectric properties was greater. For example, the dielectric constant and loss factor of samples decreased to 0.28 and 0.18 at 4.2% w.b. and 10.86 and 99.03 at 19.6% w.b. moisture content, respectively, when the frequency increased from 10 to 200 MHz at 50 °C.

### Moisture and temperature-dependent dielectric properties

**Figure 4:**
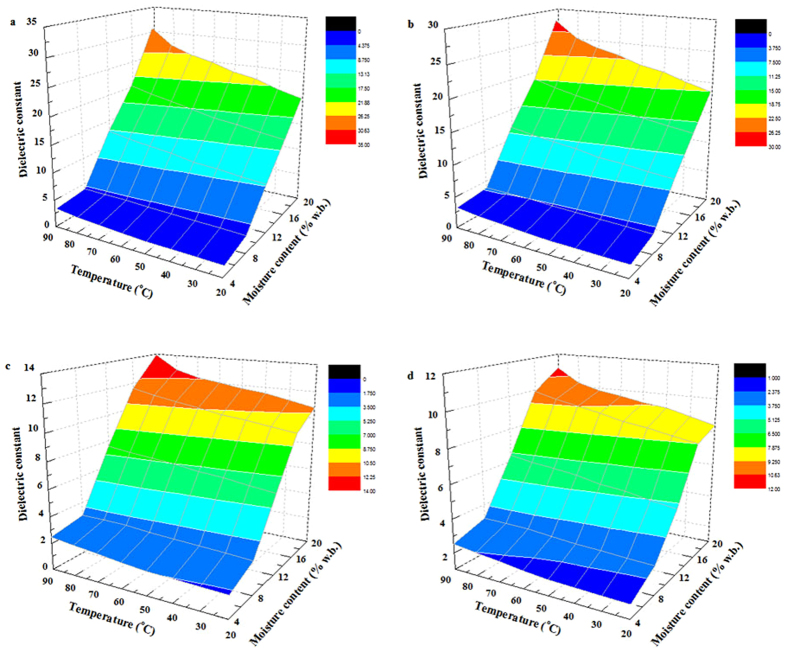
Moisture and temperature-dependent dielectric constant of almond kernels at 27 (**a**), 40 (**b**), 915 (**c**), and 2450 MHz (**d**).

**Figure 5:**
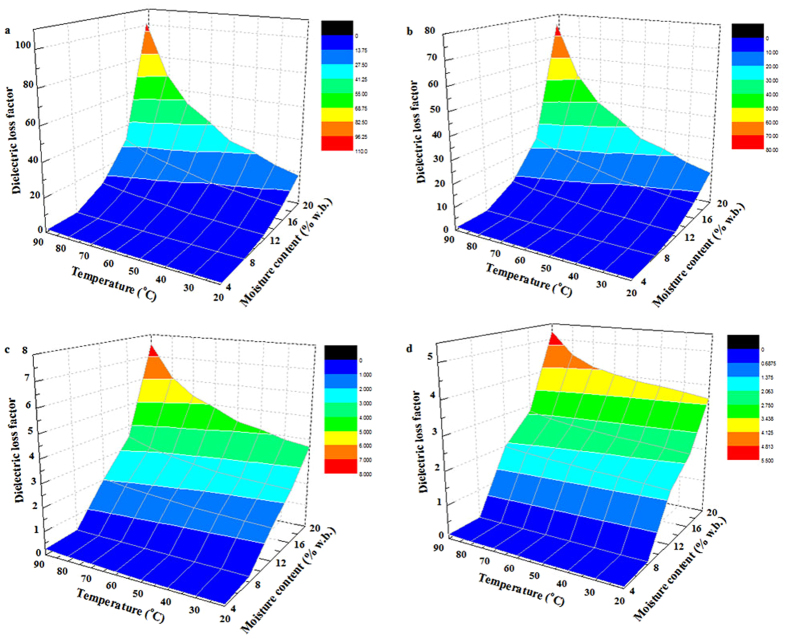
Moisture and temperature-dependent dielectric loss factor of almond kernels at 27 (**a**), 40 (**b**), 915 (**c**), and 2450 MHz (**d**).

To better understand the relationship between dielectric properties and moisture content with temperature, the moisture and temperature-dependent dielectric properties of almond kernel samples at 27, 40, 915, and 2450 MHz over a moisture content range from 4.2% to 19.6% w.b. and temperature range from 20 to 90 °C are shown in Figs 4 and 5. It was observed that both the dielectric constant and loss factor of samples increased with increasing temperature and moisture content at a certain frequency. For example, at 27 MHz for RF pasteurization, when the sample temperature increased from 20 to 90 °C, the dielectric constant increased from 1.83 to 3.25, from 20.23 to 30.79 and the dielectric loss factor increased from 0.18 to 1.23, from 18.74 to 101.42, respectively, with moisture content of 4.2% and 19.6% w.b. At 915 MHz for MW pasteurization, when the sample moisture content increased from 4.2% to 19.6% w.b., the dielectric constant increased from 1.64 to 10.77, and from 2.48 to 13.94, and the loss factor increased from 0.07 to 3.67, and from 0.26 to 7.55, respectively, at the temperature of 20 and 90 °C. Also increasing temperature from 20 to 90 °C, the dielectric constant and loss factor of almond kernels increased at 915 MHz for moisture increased from 4.2% to 19.6% w.b.

### Regression models for dielectric properties of almonds

Regression equations of dielectric constant and loss factor for almond kernels as function of temperature (20–90 °C) and moisture content (4.2–19.6% w.b.) are given at 27, 40, 915 and 2450 MHz listed in Tables 2 and 3. Regression models for dielectric constant were shown in Eqs (1–4), and regression models for dielectric loss factor were shown in Eqs (5–8). Quadratic polynomial relationships were best fit for dielectric constant and loss factor at 27, 40, 915 and 2450 MHz. ANOVA results for Eqs (1–4) and Eqs (5–8) were shown in Tables 4 and 5. All the equations provided a good fit with a coefficient of determination (R^2^) greater than 0.967 at the significance level of 0.0001 (p < 0.0001). The data suggested that the dielectric constant and loss factor for almond kernels at any given moisture content between 4.2% and 19.6% w.b. and temperature range from 20 to 90 °C at the four specific frequencies (27, 40, 915 and 2450 MHz) can be precisely predicted through these models. Also these models can be applied to estimate moisture and temperature-dependent dielectric properties used in computer simulation.

### Penetration depth

The penetration depths calculated from the obtained dielectric constant and loss factor of almond samples at four specific frequencies, five moisture levels, and eight temperatures are shown in Table 6. The penetration depth of electromagnetic wave into samples decreased with increasing frequency, moisture content, and temperature. For example, for almond samples at 60 °C with moisture content of 11.9% w.b., the penetration depth decreased from 90.62 cm to 2.74 cm when the frequency increased from 27 to 2450 MHz. The penetration depth decreased from 650.30 cm to 21.94 cm at 60 °C and 27 MHz when the moisture content of almond samples increased from 4.2% to 19.6% w.b. Moreover, the penetration depths at RF frequencies (27 and 40 MHz) were larger than those at MW frequencies (915 and 2450 MHz) for almond kernels with the same moisture contents and temperatures. For example, the range of penetration depths was from 12.79 cm to 1068.40 cm, 2.68 cm to 17.78 cm and 1.36 cm to 8.54 cm for almond kernels at 27, 915 and 2450 MHz, respectively. Smaller penetration depth at MW frequencies results in more surface heating in almond kernel. Larger penetration depths at RF frequencies mean better heating uniformity in almond kernels.

### RF heating rates of almond kernel at three moisture contents

**Figure 6:**
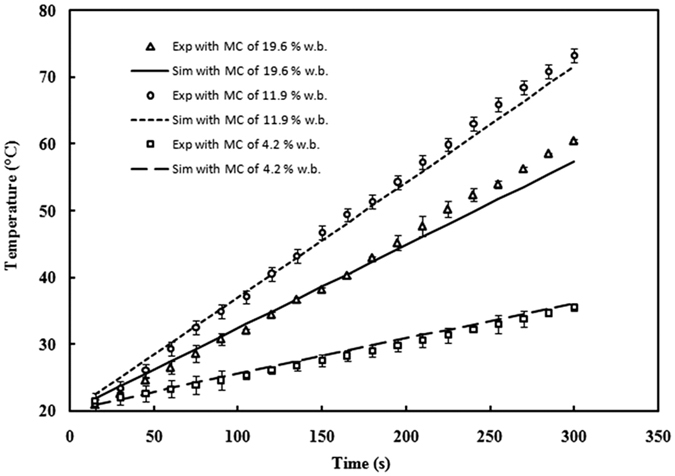
Experimental (exp with symbols) and simulated (sim with lines) temperature-time histories of almond kernels with moisture content of 4.2% (□), 11.9% (○) and 19.6% w.b. (△) when subjected to RF heating for 5 min with electrode gap of 120 mm.

The experimental and simulated temperature-time histories of almond kernels are shown in Fig. 6 with three moisture contents of 4.2%, 11.9% and 19.6% w.b. when subjected to RF heating for 5 min with electrode gap of 120 mm. It is shown that the computer simulation and experimental data agreed well with each other under three moisture levels. The sample temperatures increased almost linearly with the RF heating time but the heating rates of almond kernels with 11.9% was larger than those at 4.2% and 19.6% w.b., which were similar to those found by Huang *et al*.^28^ since increasing loss factor caused an initial increase and then a decrease in RF heating rates.

## Discussion

At the moisture content of 4.2% w.b., the peak values of dielectric constant and loss factor appeared at 2000–2800 and 500–1000 MHz are caused by lower ionic and greater bound water relaxation of almond samples at MW frequencies^18^. Similar trends have also been found in Macadamia nut and walnuts with peak values in the range of 1000–1600 and 500–1000 MHz^18^,^27^, respectively. These frequency-dependent trend differences may be caused by complex interaction among frequency, temperature, moisture and fat content^29^. However, when the moisture content was higher than 11.9% w.b., both dielectric constant and loss factor decreased with increasing frequency. Similar trends of frequency-dependent dielectric properties have been reported for nuts, wheat, beans, fruits and vegetables^18^,^30^,^31^,^32^,^33^. That’s because with higher moisture content, dipolar rotations may play a dominant effect on the dielectric properties^18^.

The dielectric constant and loss factor increased with increasing moisture content. Similar trends at a given frequency have also been found in red pepper powder, chestnut flour and macadamia nut kernels^18^,^34^,^35^. At the moisture content of 4.2% w.b., little increase in the dielectric constant and loss factor of samples was observed when the temperature ranged from 20 to 90 °C. That’s because at low moisture content, most water molecules are bound to starch or proteins in the nut kernels, which caused little ionic conduction and less free water dispersion^36^. Moreover, the dielectric constant and loss factor of almond kernels increased with increasing temperature since higher temperature reduced viscosity of biomaterials, which may raise ionic conductivity^18^.

The dielectric constant of samples determines the electric field distribution when the loss factor is far smaller than dielectric constant. Therefore, optimum RF heating uniformity in almond kernels could be achieved using a container material with similar dielectric constant to the sample^28^. For example, Huang *et al*.^37^ improved the RF heating uniformity of dry soybean samples by polystyrene container compared to polypropylene one through both experiment and computer simulation. The corn samples in the polyetherimide container had a better RF heating uniformity due to close dielectric constant between polyetherimide container and corn^38^. The dielectric constant of surrounding material can be predicted according to the following regression equation:


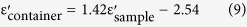


Where 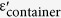 is the dielectric constant of the surrounding material and 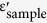 is the dielectric constant of the sample. Thus, to improve the RF heating uniformity, appropriate surrounding material should be chosen according to dielectric constant of almond kernels and the above equation^39^.

Loss factor measures the energy dissipated in the materials from the applied electric field^29^. The higher the loss factor, the higher rate of temperature increases. The behaviour of decreasing moisture content caused low loss factor at RF and MW frequencies may lead to low sample temperature, which is potential drying advantages, referred to “moisture levelling effect”^40^,^41^. During RF or MW heating, almond kernels with high moisture content absorb more energy, and thus heated preferentially and more rapidly, causing more water vaporization. Since the vapour pressure gradient could be developed from the kernel centre to the surface, surface hot air heating in combination with internal RF and MW heating might be an appropriate way to improve drying efficiency. The higher sample temperature resulted in larger loss factor, this phenomenon is referred to “thermal run away” during pasteurization of almond kernels using RF or MW energy^42^,^43^. The temperature distributions need to be uniform before and during the RF or MW heating.

Due to larger penetration depths at RF ranges than at MW frequencies for almond kernel with the same moisture content and temperature, the uniformity and throughput under RF heating could be better than MW heating, providing practical large scale treatments for pasteurization of almond kernels.

## Methods

### Materials

The variety of almond kernel used in this study was “Nonpareil”, which was purchased from Paramount Farming Company (Modesto, CA, USA). The almond kernel was sealed into polyethylene bags and stored at 4 ± 1 °C until testing. The initial moisture content (MC) and water activity (Aw) of almond kernels were 4.18 ± 0.02% w.b. and 0.47, respectively.

### Sample preparation

To prepare almond samples with five needed moisture content levels (4.2, 8.0, 11.9, 15.8, and 19.6% w.b.) for dielectric properties measurement, 200 g of almond kernels with the original moisture content (4.2% w.b.) were placed in 5 prepared plastic bottles. Predetermined amounts of distilled water were sprayed on the almond kernels. Then, the bottles were tightly capped and placed in a refrigerator at 4 °C for 7 d. During this period, they were thoroughly mixed three times a day to ensure the uniformity of moisture distribution.

### Moisture content, water activity and density measurement

MC of almond kernel was determined according to the AOAC Official Method 925.40. Aw of almond kernel was determined by a water activity meter (Aqua Lab 4TE, Decagon Devices, Inc., Pullman, WA, USA). The true density of almond kernels at different moisture content was determined at room temperature using the liquid displacement method. Toluene (C_7_H_8_) was used as the displacement liquid due to low surface tension^44^ and little absorption by almond kernels. Detailed measurement procedures can be found by others^16^,^17^,^45^.

### Dielectric properties measurement

**Figure 7:**
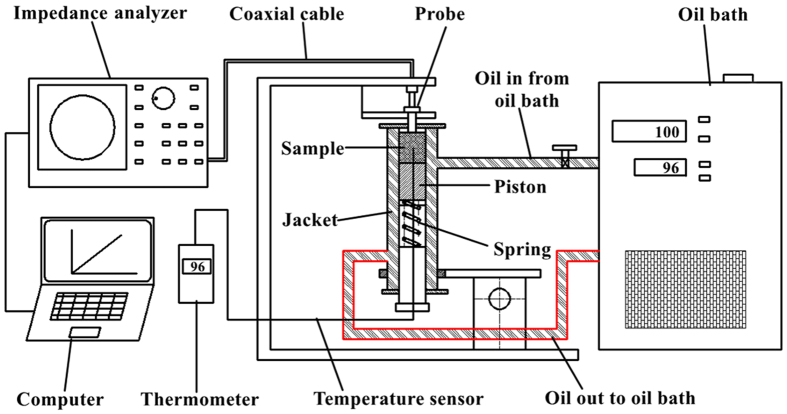
Schematic diagram of the dielectric properties measurement system.

The open-ended coaxial probe method is widely used to measure dielectric properties of many materials over a broad frequency range due to its easy operation and high accuracy. It is usually used to measure liquid or semi-solid materials. However, for irregularly shaped nut kernel, ground samples of the nut were used for measuring dielectric properties since they can be closely contacted to flat tip of the probe^16^,^17^,^18^,^23^. Dielectric properties of ground almond kernels were measured in two replicates at 20, 30, 40, 50, 60, 70, 80, and 90 °C between 10 and 3000 MHz using an open-ended coaxial probe system (Fig. 7). This system consisted of an impedance analyzer (E4991B-300, Keysight Technologies Co. LTD., Palo Alto, California, USA) with a calibration kit (E4991B-010), a high-temperature coaxial cable, the coaxial probe with keysight dielectric probe kit (85070E-020), a custom-built sample test holder, an oil circulated bath (SST-20, Guanya Constant Temperature Cooling Technology Co. LTD., Wuxi, China) and a computer. Before the measurement started, the impedance analyzer and auxiliary computer were turned on and kept in a standby condition for at least 30 min. Then the E4991B calibration kit was used to calibrate the coaxial probe with open, short and 50 Ω resistance in order. The impedance analyzer system was calibrated by air, short and 25 °C distilled water in sequence. Once the system was calibrated, the samples were put into custom-built sample test holder (21 mm in diameter and 50 mm in height) and confined with a pressure spring to ensure a close contact between the tip of the coaxial probe and the sample during the measurements. The sample MC was maintained during the measurements because the sample holder was air tight. The sample temperature was controlled by circulating oil from an oil bath into the jacket of the test holder. The sample temperature during the measurement was monitored by a thermocouple (HH-25TC, Type-T, OMEGA Engineering Inc., Stamford, Connecticut, USA). After each measurement, the probe and the sample holder were cleaned with deionized water and wiped dry.

### Power penetration depth

The penetration depth (*d*_*p*_, m) of RF and MW power in a material is defined as the distance in meters where the power is reduced to 1/*e (e* = 2.718) of the power passing the surface^46^. It can be calculated as follows:


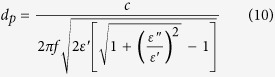


Where *c* is the speed of light in free space (3 × 10^8^ m s^−1^) and *f* is the frequency (Hz).

### Statistical analysis

Mean and standard deviations were calculated from the two replicates. Analysis of variance (ANOVA) was used to determine whether temperature and moisture content had significant influences on the dielectric properties of almond kernels.

### RF heating systems and measurement of temperature

The relationship between dielectric properties and RF heating was explained by determining the heating rate of almond kernel through both experiment and computer simulation. A 6 kW, 27.12 MHz free-running oscillator RF system (SO6B, Strayfield International, Workingham, U.K.) with an electrode gap of 120 mm and heating time of 5 min was used to heat 2.4 kg almond kernel in a plastic container (300 mm × 220 mm × 60 mm). This container was vertically separated into three equal sections by two pieces of thin plastic film according to three moisture levels (4.2%, 11.9%, and 19.6% w.b.). Then it was placed at the centre of the bottom electrode, which was the same as Zhang *et al*.^17^. The six-channel fibre optical temperature sensor system (HQ-FTS-D120, Heqi Technologies Inc., Xian, China) with an accuracy of ±0.5 °C was used for measuring the centre temperature of almond kernel. Commercialized software COMSOL Multiphysics (v4.3a, CnTech Co., LTD., Wuhan, China) based on finite element method (FEM) was used to simulate the RF heating process for almond kernel using a computer workstation (Intel^®^ Core™ i5-2400 CPU, 8 GB memory, 64-bit Windows 10 Professional operating system). The electric potential on the top electrode was considered as 5300–5800 V based on the measured anode current, which was described by Huang *et al*.^28^. The initial temperature of the air, plastic container, almond, upper and bottom electrodes was set at room temperature (20 °C). Electrical insulation 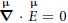 was considered for the external walls of the RF cavity. The convective heat transfer boundary conditions were assigned at the outer sample surfaces exposed to air with heat transfer coefficient of *h* = 20 Wm^−2^ K^−1^. Almond kernel properties, including thermal, physical and dielectric properties, were assumed homogeneous and isotropic, and the density of almond kernels was assumed as a function of temperature. The mesh size was chosen based on the convergence study when the difference of the maximum temperature between successive calculations was less than 0.1%. The direct linear system solver (UMFPACK) was used with a relative tolerance and absolute tolerance of 0.01 and 0.001, respectively, with the initial and maximum time steps of 0.001 s and 0.1 s. The simulated sample temperature was further used to confirm the experimental data.

## Additional Information

**How to cite this article**: Li, R. *et al*. Dielectric properties of almond kernels associated with radio frequency and microwave pasteurization. *Sci. Rep.* **7**, 42452; doi: 10.1038/srep42452 (2017).

**Publisher's note:** Springer Nature remains neutral with regard to jurisdictional claims in published maps and institutional affiliations.

## Figures and Tables

**Table 1:** True densities of almond kernels at five moisture contents.

Moisture content (%, w.b.)	Density ± SD (g cm^−3^)
4.2	1.039 ± 0.007
8.0	1.041 ± 0.003
11.9	1.044 ± 0.008
15.8	1.049 ± 0.013
19.6	1.060 ± 0.008

**Table 2:** Regression equations of dielectric constant as a function of temperature (*T*, 20–90 °C) and moisture content (*M*, 4.2–19.6% w.b.) of almond samples.

Frequency (MHz)	Dielectric constant (*ɛ*′)
27	*ε*′ = 2.96 − 0.09*T* + 0.05*M* + 6.56 × 10^−3^*TM* + 2.41 × 10^−3^*T*^2^ − 0.07*M*^2^ − 2.31 × 10^−4^*T*^2^*M* + 6.37 × 10^−4^*TM*^2^ − 1.65 × 10^−5^*T*^3^ + 0.01*M*^3^ + 3.72 × 10^−6^*T*^2^*M*^2^ + 8.94 × 10^−7^*T*^3^*M* − 2.79 × 10^−5^*TM*^3^ + 3.79 × 10^−8^*T*^4^ − 3.12 × 10^−4^*M*^4^ (1)
40	*ε*′ = 25.72 − 0.21*T* − 10.12*M* + 0.02*TM* + 5.35 × 10^−3^*T*^2^ + 1.46*M*^2^ − 4.24 × 10^−4^*T*^2^*M* + 5.26 × 10^−4^*TM*^2^ − 4.45 × 10^−5^*T*^3^ − 0.08*M*^3^ + 7.18 × 10^−6^*T*^2^*M*^2^ + 1.74 × 10^−6^*T*^3^*M* − 2.31 × 10^−5^*TM*^3^ + 1.39 × 10^−7^*T*^4^ + 1.59 × 10^−3^*M*^4^ (2)
915	*ε*′ = 11.67 − 0.12*T* − 3.99*M* + 0.01*TM* + 2.69 × 10^−3^*T*^2^ + 0.52*M*^2^ − 2.92 × 10^−4^*T*^2^*M* + 3.10 × 10^−4^*TM*^2^ − 1.72 × 10^−5^*T*^3^ − 0.02*M*^3^ + 5.26 × 10^−6^*T*^2^*M*^2^ + 1.10 × 10^−6^*T*^3^*M* − 2.17 × 10^−5^*TM*^3^ + 3.48 × 10^−8^*T*^4^ + 3.58 × 10^−4^*M*^4^ (3)
2450	*ε*′ = 2.96 − 0.10*T* + 0.05*M* + 6.56 × 10^−3^*TM* + 2.41 × 10^−3^*T*^2^ − 0.07*M*^2^ − 2.31 × 10^−4^*T*^2^*M* + 6.37 × 10^−4^*TM*^2^ − 1.65 × 10^−5^*T*^3^ + 0.01*M*^3^ + 3.72 × 10^−6^*T*^2^*M*^2^ + 8.94 × 10^−7^*T*^3^*M* − 2.79 × 10^−5^*TM*^3^ + 3.79 × 10^−8^*T*^4^ − 3.12 × 10^−4^*M*^4^ (4)

**Table 3:** Regression equations of dielectric loss factor as a function of temperature (*T*, 20–90 °C) and moisture content (*M*, 4.2–19.6% w.b.) of almond samples.

Frequency (MHz)	Dielectric loss factor (*ɛ*″)
27	*ε*″ = 83.84 − 3.60*T* − 22.89*M* + 0.58*TM* + 0.07*T*^2^ + 2.33*M*^2^ − 5.76 × 10^−3^*T*^2^*M* − 0.03*TM*^2^ − 5.78 × 10^−4^*T*^3^ − 0.10*M*^3^ + 1.51 × 10^−4^*T*^2^*M*^2^ + 1.93 × 10^−5^*T*^3^*M* + 7.11 × 10^−4^*TM*^3^ + 1.96 × 10^−6^*T*^4^ + 1.73 × 10^−3^*M*^4^ (5)
40	*ε*″ = 60.82 − 2.60*T* − 16.64*M* + 0.42*TM* + 0.05*T*^2^ + 1.70*M*^2^ − 4.16 × 10^−3^*T*^2^*M* − 0.02*TM*^2^ − 4.19 × 10^−4^*T*^3^ − 0.08*M*^3^ + 1.08 × 10^−4^*T*^2^*M*^2^ + 1.40 × 10^−5^*T*^3^*M* + 5.06 × 10^−4^*TM*^3^ + 1.42 × 10^−6^*T*^4^ + 1.28 × 10^−3^*M*^4^ (6)
915	*ε*″ = 9.38 − 0.15*T* − 3.69*M* + 0.02*TM* + 2.83 × 10^−3^*T*^2^ + 0.51*M*^2^ − 2.47 × 10^−4^*T*^2^*M* − 1.38 × 10^−3^*TM*^2^ − 2.43 × 10^−5^*T*^3^ − 0.03*M*^3^ + 6.31 × 10^−6^*T*^2^*M*^2^ + 8.68 × 10^−7^*T*^3^*M* + 2.89 × 10^−5^*TM*^3^ + 8.04 × 10^−8^*T*^4^ + 5.53 × 10^−4^*M*^4^ (7)
2450	*ε*″ = 11.54 − 0.09*T* − 4.95*M* + 0.01*TM* + 1.65 × 10^−3^*T*^2^ + 0.72*M*^2^ − 1.58 × 10^−4^*T*^2^*M* − 5.29 × 10^−4^*TM*^2^ − 1.35 × 10^−5^*T*^3^ − 0.04*M*^3^ + 3.36 × 10^−6^*T*^2^*M*^2^ + 6.00 × 10^−7^*T*^3^*M* + 7.66 × 10^−6^*TM*^3^ + 4.12 × 10^−8^*T*^4^ + 8.36 × 10^−4^*M*^4^ (8)

**Table 4:** Significance of probability of regressed models of Eqs (1–4) for almonds at four frequencies of interest.

Variance and R^2^	27 MHz (Eq. 1)	40 MHz (Eq. 2)	915 MHz (Eq. 3)	2450 MHz (Eq. 4)
*T*	<0.0001	<0.0001	<0.0001	<0.0001
*M*	<0.0001	<0.0001	<0.0001	<0.0001
*TM*	<0.0001	<0.0001	<0.0001	<0.0001
*T*^*2*^	0.4180	0.8230	0.4750	0.4180
*M*^*2*^	<0.0001	<0.0001	<0.0001	<0.0001
*T*^*2*^*M*	0.2523	0.0155	0.0081	0.2523
*TM*^*2*^	0.0229	<0.0001	<0.0001	0.0229
*T*^*3*^	0.0769	0.0851	0.0307	0.0769
*M*^*3*^	<0.0001	<0.0001	<0.0001	<0.0001
*T*^*2*^*M*^*2*^	0.0012	0.0215	0.0001	0.0012
*T*^*3*^*M*	0.0012	0.0207	0.0006	0.0012
*TM*^*3*^	<0.0001	0.1348	0.0013	<0.0001
*T*^*4*^	0.6251	0.5333	0.6967	0.6251
*M*^*4*^	<0.0001	<0.0001	<0.0001	<0.0001
Model	<0.0001	<0.0001	<0.0001	<0.0001
R^2^	0.9978	0.9974	0.9983	0.9978

**Table 5:** Significance of probability of regressed models of Eqs (5–8) for almond kernels at four frequencies of interest.

Variance and R^2^	27 MHz (Eq. 5)	40 MHz (Eq. 6)	915 MHz (Eq. 7)	2450 MHz (Eq. 8)
*T*	0.7831	0.9175	0.2028	0.2770
*M*	<0.0001	<0.0001	<0.0001	<0.0001
*TM*	0.3507	0.2828	0.1612	0.3065
*T*^*2*^	0.3838	0.3977	0.6945	0.4505
*M*^*2*^	0.0985	0.0809	<0.0001	<0.0001
*T*^*2*^*M*	<0.0001	<0.0001	<0.0001	<0.0001
*TM*^*2*^	<0.0001	<0.0001	<0.0001	<0.0001
*T*^*3*^	0.0205	0.0204	0.0229	0.0049
*M*^*3*^	<0.0001	<0.0001	0.6310	<0.0001
*T*^*2*^*M*^*2*^	<0.0001	<0.0001	<0.0001	<0.0001
*T*^*3*^*M*	0.0042	0.0042	0.0052	0.0011
*TM*^*3*^	<0.0001	<0.0001	<0.0001	0.0355
*T*^*4*^	0.3152	0.3179	0.3726	0.4258
*M*^*4*^	0.0956	0.0915	<0.0001	<0.0001
Model	<0.0001	<0.0001	<0.0001	<0.0001
R^2^	0.9668	0.9687	0.9920	0.9958

**Table 6:** Penetration depth (cm) of electromagnetic wave into samples calculated from the measured dielectric properties of almond kernels at four specific frequencies over three temperatures and five moisture contents.

Moisture content (% w.b.)	T (°C)	Penetration depth (cm)			
		27 MHz	40 MHz	915 MHz	2450 MHz
4.2	20	1068.40 ± 55.21	743.93 ± 45.70	17.78 ± 1.80	8.54 ± 0.89
	60	725.91 ± 28.31	520.78 ± 38.47	15.90 ± 1.17	7.58 ± 0.65
	90	650.30 ± 47.97	394.75 ± 21.60	13.54 ± 1.85	6.06 ± 0.28
8.0	20	436.52 ± 8.73	242.55 ± 17.40	12.18 ± 0.44	5.70 ± 0.97
	60	259.79 ± 1.83	142.84 ± 2.92	10.74 ± 0.24	4.19 ± 0.75
	90	231.22 ± 1.96	114.76 ± 1.55	9.95 ± 0.64	3.83 ± 0.89
11.9	20	187.44 ± 2.73	90.92 ± 1.73	9.21 ± 0.17	2.85 ± 0.52
	60	90.62 ± 1.71	77.81 ± 0.62	8.33 ± 0.01	2.74 ± 0.39
	90	56.95 ± 0.93	49.10 ± 0.65	7.08 ± 0.16	2.57 ± 0.22
15.8	20	69.18 ± 0.14	59.34 ± 0.16	6.92 ± 0.42	2.45 ± 0.09
	60	42.69 ± 0.82	36.90 ± 0.61	6.35 ± 0.81	2.37 ± 0.07
	90	26.82 ± 0.40	23.34 ± 0.92	5.11 ± 0.28	2.22 ± 0.03
19.6	20	40.41 ± 0.32	34.76 ± 0.89	4.71 ± 0.18	1.67 ± 0.03
	60	21.94 ± 0.59	19.00 ± 0.25	3.78 ± 0.21	1.61 ± 0.06
	90	12.79 ± 0.97	11.05 ± 0.73	2.68 ± 0.28	1.36 ± 0.08
